# Augmented or Mixed Reality Enhanced Head-Mounted Display Navigation for In Vivo Spine Surgery: A Systematic Review of Clinical Outcomes

**DOI:** 10.3390/jcm12113788

**Published:** 2023-05-31

**Authors:** Kristóf Móga, Áron Hölgyesi, Zsombor Zrubka, Márta Péntek, Tamás Haidegger

**Affiliations:** 1 Doctoral School of Theoretical and Translational Medicine, Semmelweis University, 1085 Budapest, Hungary; 2 Antal Bejczy Center for Intelligent Robotics (BARK), Óbuda University, 1034 Budapest, Hungary; 3 Health Economics Research Center (HECON), University Research and Innovation Center (EKIK), Óbuda University, 1034 Budapest, Hungary; 4 Austrian Center for Medical Innovation and Technology (ACMIT), 2700 Wiener Neustadt, Austria; 5 University Research and Innovation Center (EKIK), Óbuda University, 1034 Budapest, Hungary

**Keywords:** spine surgery, pedicle screw placement, augmented reality, mixed reality, navigation and robotics in surgery

## Abstract

Background: This research paper provides a systematic literature review (SLR) on the current status of augmented-reality head-mounted devices (AR-HMDs) that guide and navigate spine surgeries and pedicle screw placement. Methods: Embase, Scopus, PubMed, Cochrane Library and IEEE Xplore databases were screened for the systematic literature search to collect and statistically analyze live patient clinical, procedural and user experience data. Multi-level Poisson and binominal models were used for analysis. Results: In vivo patient data, only the clinically widely used Gertzbein–Robbins Scale, were published as an outcome in the recent heterogeneous literature. The statistical analysis supports the hypothesis that using AR-HMDs has the same clinical outcomes as using more expensive robot-assisted surgical (RAS) systems. Conclusions: AR-HMD-guided pedicle screw insertion is reaching its technology readiness, providing similar benefits to RAS. Further meta-analysis is expected in the future from higher case-numbered and standardized randomized clinical trials.

## 1. Introduction

The recent medical and surgical advancements of image guided surgery (IGS) and computer integrated surgery (CIS) have reached spine surgery as well [1,2,3,4,5]. Intelligent imaging technologies, such as augmented reality (AR) or mixed reality (MR) enhanced navigation can support various spine surgery interventions, including vertebroplasty, kyphoplasty or tumor surgery. Nonetheless, AR/MR enhanced navigation has been most used in pedicle screw (PS) placement. IGS naturally supports PS placement with intra-operative navigation, which can be further leveraged by enhancing the registration and control of both robotic and AR/MR systems [6].

The collective term of extended reality involves three main dimensions of how digital information can be applied as an addition to the real environment. Virtual reality (VR) encloses all the computer-generated information in a headset for the user, making them unable to interact with the real environment, which is why the solution is mainly aimed at gaming purposes. AR and MR are different technologies in complexity that enable the user to interact with the virtual content without canceling out the physical world through a transparent head-mounted display (HMD). A slight difference between the two is that AR technology overlays the digital content (text, images, etc.) to the world, but MR allows the user to interact with these (such as 3D holographic models [7]). The authors recognized through the literature search that using the two terms in the medical domain is not 100% consistent and is frequently mixed up.

**Figure 1:**
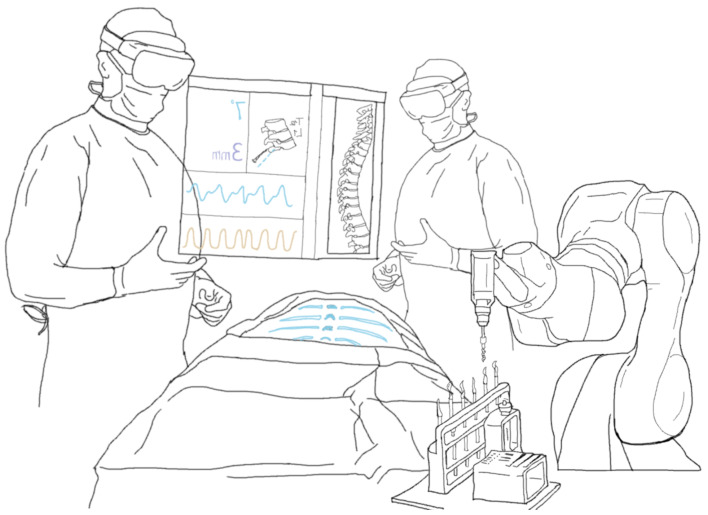
The concept of augmented reality head-mounted-device navigated semi-autonomous robotic spine surgery [15].

Nowadays, still only a few available AR/MR solutions can be found for intra-operative use, mainly not even commercially available systems, just prototypes. However, as the technology is constantly and rapidly advancing, it is becoming clearer that augmented reality and mixed reality navigation can allow safer and more accurate navigation and guidance in the field of spine surgery [8]. Using a system such as xVision by Augmedics (the most widely used and only FDA-approved navigation known by the authors till the publishing date), the guidance is based on pre-operative diagnostic CT or MRI scan images and intra-operative cone-beam CT scans. These examinations can be segmented into 3D models manually, semi-automatically or via artificial intelligence (AI) and deep-learning-driven methods through image computing platforms (e.g., 3D Slicer [9,10]). Those models can be projected as monitor based, microscope based, or HMD AR or MR holograms [7] to the surgical site after the image–patient registration procedure using infrared cameras or electromagnetic tracking. The preference for the see-through AR-HMD navigation is that the holographic model is projected into the user’s (surgeon’s) field of view, preventing the further disruption of the surgical workflow. This could decrease the procedures’ overall time and altogether lower the risk of more prolonged anesthesia (Figure 1).

One of the most popular medical fields where AR and MR are used is spine surgery and pedicle screw placement. PSs are used for stabilizing potential (such as degenerative spinal diseases and spinal stenosis) and existing instabilities (such as post-laminectomy syndrome and pseudoarthrosis) of the spinal column, as well as spinal traumas and fractures, tumor surgeries and spinal deformities (such as kyphoscoliosis) [11,12]. The widely used free-handed (FH) percutaneous PS placement procedure involves the insertion of pedicle screws into the pre-formed bone pathways and fixating the spine segment with metal rods with 2D fluoroscopy images as guidance. Despite the advantages of minimal invasive—percutaneous—PS placement (e.g., smaller incisions, less pain and faster recovery), with the best intent for patient care, a meta-analysis by Staartjes et al. showed a 3.3% need for secondary revision surgery because of the misplaced screw implantation of FH spine fixations. Using new technologies could decrease the incidence of misplacement complications, leading to a reduction in patient morbidity and mortality, reducing the cost of care USD 23,865–32,915per revision surgery [13,14].

The effect of HMD-based navigation on screw implantation accuracy, operating time, overall radiation dose and cost benefits are not yet proven because of the low number and heterogeneous literature discussing these data. A systematic literature review in 2020 by Bursröm et al. gathered and analyzed 28 articles focusing on AR navigation in spine surgery. Still, limited clinical data were presented, and meta-analysis could not be performed [16]. As the technology is rapidly advancing and new FDA (USA Food and Drug Administration) approved AR-capable navigation HMD systems are being used lately, the re-screening of the literature has become necessary.

The authors aimed to systematically review the literature for living human studies reporting clinical outcomes (including procedural and user experience data) with AR/MR–HMDs used for navigation in spine surgery. For the period up to 27 November 2020, we relied on the systematic literature review by Bursröm et al. based on their search in PubMed and Web of Science databases. Our focus is on the detailed analysis of studies that have been published since then. Secondarily, we investigated whether these more recent studies allow the meta-analysis of the available data, and whether these confirm or contradict the existing knowledge.

## 2. Materials and Methods

### 2.1. Search Strategy

This review is following the PRISMA guidelines, but was not registered. The search of the systematic literature was performed for the period between 27 November 2020 and 1 May 2023. The search was designed to identify articles in which AR- or MR-guided (in HMD form) spine surgery was used in humans for navigation purposes. PubMed, Scopus, Embase, Cochrane Library and IEEE Xplore databases were screened. The following keywords were applied: “augmented reality”, “mixed reality” and “spine surgery”. The exact search terms are presented in the Appendix A.

### 2.2. Eligibility Criteria

#### 2.2.1. Patients

Only studies involving human patients with a minimum sample size of five patients or a minimum of five implanted screws were included.

#### 2.2.2. Intervention

AR/MR-HMD navigation in PS spine surgery.

#### 2.2.3. Comparator

Any.

#### 2.2.4. Outcome

Outcomes comprise any clinical outcome (e.g., recovery rates, length of hospitalization, visual analogue scale, and post-operative follow-up), accuracy data (linear tip error, angular trajectory error, and Gertzbein–Robbins scale), complications, procedural data (operating time and radiation dose) and user experience measured by any standard validated method.

#### 2.2.5. Setting

Both experimental and non-experimental settings were included.

#### 2.2.6. Publication Types

Articles written in English were included. The authors excluded research papers describing teleconsultation, telemedicine and educational use of AR and MR technologies, any abstracts, opinions, letters, reviews, SLRs, conference papers and single case reports.

### 2.3. Screening

Records identified during the search were screened in two steps. First, the hits were screened by their title and abstract by two reviewers (KM and AH) independently. Disagreements were solved by discussions and wherever it was needed, a third researcher was involved. Second, the selected articles were downloaded in full text and screened by applying the eligibility criteria, using the same independent review method.

### 2.4. Data Extraction

From the included articles, clinical, procedural and accuracy data were collected, analyzed and narrated by one reviewer (KM). Data extraction included the number of patients who went over spine surgery, the number and accuracy of inserted pedicle screws with accuracy data such as the Gertzbein–Robbins scale (GRS), linear tip error (LTE) and angular trajectory error (ATE), operating time (OT) and surgical complications as the occurrence of specific intra-operative and post-operative ones. Demographic data and the proportion of affected patients were analyzed as well.

The user experience of surgeons and criticism about HMDs and AR/MR navigation were collected too, highlighting the presence of situation awareness, technical challenges and limitations of the different manufactured HMDs. As secondary data, where those were available, the region of spine operation (collar, thoracic, lumbar or sacral), disease etiology (trauma, compression fracture and tumor) and patient-reported outcomes were all gathered.

### 2.5. Analyses

A descriptive analysis of included studies was performed. Studies fulfilling our eligibility criteria in the systematic review by Bursröm et al. were analyzed to assess whether those together with the more recent studies were suitable for analysis. For this, multi-level Poisson and multi-level binomial models were used.

Statistical analysis and meta-analysis were performed with STATA 17. This SLR followed the preferred reporting items on systematic reviews and meta-analysis (PRISMA) guidelines [17].

## 3. Results

**Figure 2:**
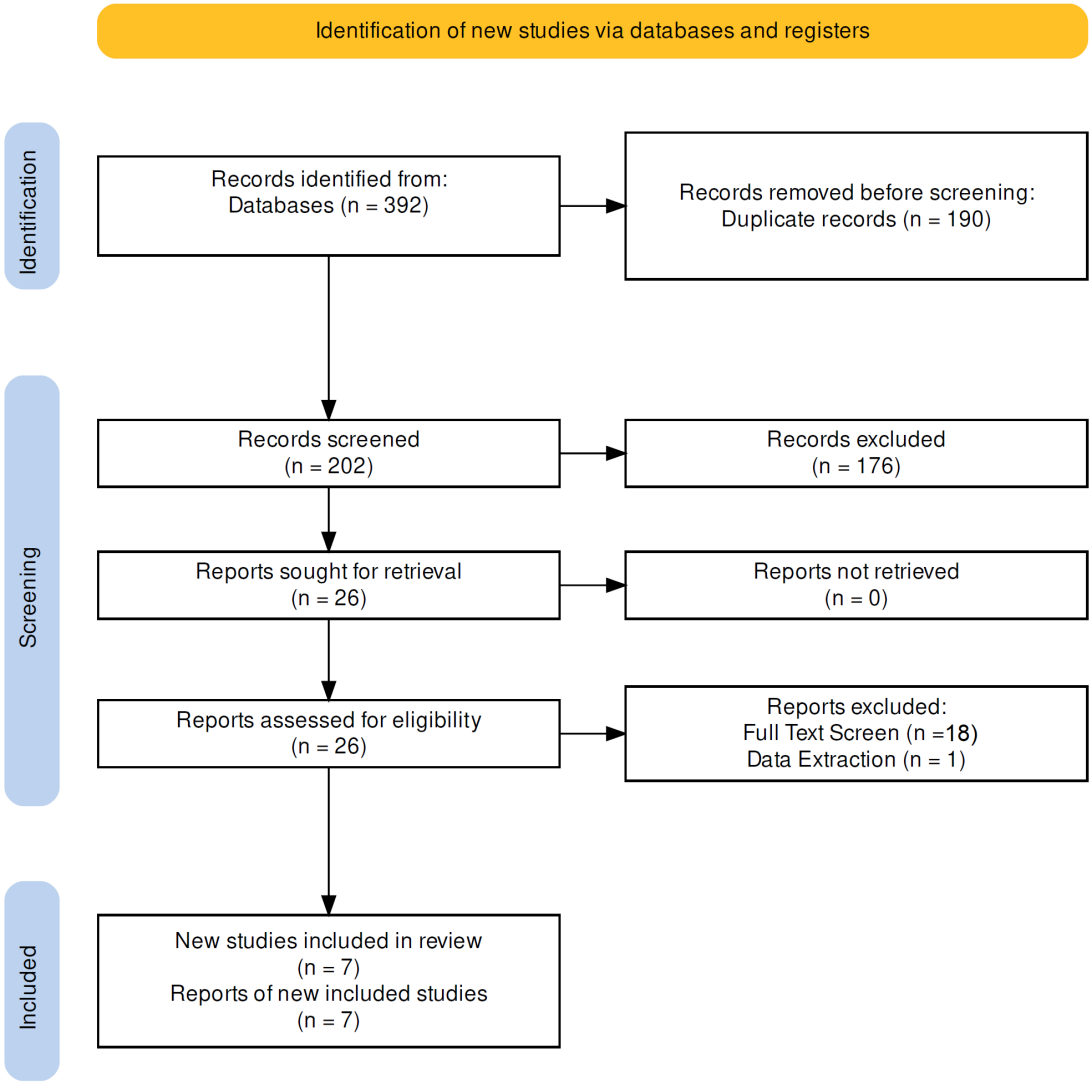
PRISMA chart of literature search.

From the previous systematic literature review by Bursröm et al., one article fulfilled our eligibility criteria [18]. From the new search time period of 27 November 2020 to 1 May 2023, 392 publications were identified. With Mendeley Desktop 1.19.8 software [19] 190 duplicates were removed, and 202 records were screened by title and abstract. During this screening, 176 articles were excluded, as those were not eligible for inclusion criteria. The full-text review was performed on the remaining 26 papers, where 19 publications were excluded, as those did not contain valid clinical outcome data on live patients. Altogether, 7 articles were included in the analyses. Figure 1 contains a PRISMA flow diagram about the search and screening (Figure 2) [17].

### 3.1. General Data and Patient Demographics

Table 1 contains the patients’ demographic and general data. Overall, 272 patients (47% male and 53% female) went through PS insertion using AR/MR-HMD navigation, with a weighted average age of 59.34 years. Patients’ average body mass index (BMI) was reported as 30.34 kg/m^2^. Only one article added information about patients’ pre-operative conditions as the American Society of Anesthesiologists score (96.9% ASA 2 and 3) and Charlson Comorbidity Index (CCI, 0.3 ± 0.5) [20]. The disease etiology and PS-insertion indications are the following:

- Existing instability (pseudoarthrosis and post-laminectomy): 16 patients (6.15%);
- Potential instability (Stenosis, Degenerative): 200 patients (76.92%);
- Trauma, unstable fracture: 8 patients (3.07%);
- Tumor, infection: 14 patients (5.38%);
- Deformity: 22 patients (8.46%).

**Table 1:** General and demographic data of patients operated with AR/MR-HMD (Augmented-/Mixed Reality Head-Mounted Display) navigation. N/A Not Added. PS Pedicle Screw insertion.

Author	Patients Nr.	Age (Average in Years)	BMI	Disease Etiology	Procedure	Surgeon	Spine Segment	Surgical Complication
Li, J. et al. [21]	7 (N/A)	N/A	N/A	fracture (n = 7, 100%)	PS, decompression, rod fixation	1 senior	Lumbar	0
Liu, A. et al. [22]	28 (11 Male/17 Female)	62.5 ± 13.8	31 ± 8.6	degenerative (n = 12, 43%); deformity (n = 12, 43%); tumor (n = 3, 11%); trauma (n = 1, 4%)	PS, osteotomy, discectomy, interbody placement, corpectomy, tumor resection	3 seniors	Thoracic (33%), Lumbar (55%) and Sacral 1 (13%)	0
Yahanda, A.T. et al. [23]	9 (5 Male/4 Female)	71.9 ± 11.5	27.3 ± 5.6	spinal tumor (n = 4, 44.4%); degenerative (n = 3, 33.3%); deformity (n = 1, 11.1%); deformity and infection (n = 1, 11.1%)	PS	1 specialist	Thoracic (50.8%), Lumbar (49.2%)	0
Bhatt, F.R. et al. [20]	32 (13 Male/19 Female)	50.9 ± 15.0	30.3 ± 4.9	stenosis (n = 10, 31.3%); lumbar post-laminectomy syndrome (n = 9, 28.1%); deformity (n = 6, 18.8%); instability (n = 5, 15.6%); pseudoarthrosis (n = 2, 6.3%)	PS and cortical screw insertion	3 specialists	Thoracic, Lumbar	0
Butler, A.J. et al. [24]	165 (83 Male/82 Female)	59.74	30.80	degenerative (n = 156, (94.5%); tumour (n = 6, 3.5%); deformity (n = 3, 1.8%)	Transforaminal interbody fusion, Lateral lumbar interbody fusion, Anterior lumbar interbody fusion, Combined interbody technique, Stabilization (83 cases-50.3%-single level, 55 cases-33.3%-2-level, 18 cases-10.9%-3-level, 9 cases-5.4%-greater than 3-levels)	3 seniors	Lumbar (97.3%), Thoracic (2.7%)	0
Harel, R. et al. [25]	19 (8 Male/11 Female)	59.52 ± 12.49	26.98 ± 3.58	spondylosis (n = 19, 100%)	PS	6 senior	Lumbar, Sacral	0
Judy, B.F. et al. [26]	12 (N/A)	N/A	N/A	deformity, degenerative disease and tumour	S2AI screw placement	2 seniors	Sacral	0

Before the PS insertion, the most common to the rarest symptoms were lumbar back pain, radicular pain, weakness of limb, loss of sensation and urinary retention. The pre-operative visual analog scale (VAS) and Oswestry disability index (ODI) were measured as an average of VAS 6.7 ± 1.8 and ODI 82.7 ± 6.2. All the papers added information about the operation-performing surgeons, who were trauma/orthopedics specialists or senior (chief) medical doctors. The pedicle screw fixations were performed on the lumbar, thoracic, thoraco-lumbar and Sacral 1 vertebrae.

### 3.2. Accuracy and Tracking

Remarkable data description heterogeneity was recognized through the literature review. A major reason could be the lack of standardized requirements for data publication in the field of spine surgery or, more precisely, AR-navigated spine surgery. On this basis, no information was identified in the four analyzed articles regarding linear tip error (LTA) and angular trajectory error (ATE). However, the clinically widely used Gertzbein–Robbins (GRS) classification score was presented in all of the screened papers based on intra-operative C-, O-arm or CT scans. GRS has 5 grades from A to E, based on the pedicle cortex breaching of the implanted screws [27]:

- A—no breach detected in intrapedicular screw position;
- B—screw exceeding the pedicle cortex is maximum 2 mm;
- C—screw exceeding the pedicle cortex is 2–4 mm;
- D—screw exceeding the pedicle cortex is 4–6 mm;
- E—screw exceeding more than 6 mm or outside of the pedicle.

Only Grades A and B can be considered satisfactory operation results, as in cases C to E, mild-to-severe neurological symptoms could occur during the post-operative follow-up [28].

Table 2 shows the clinical accuracy data of the reviewed publications. Altogether, 1258 pedicle and cortical screws were implanted into the 272 patients, an average of 4.6 screws/patient with a range of 2–15. The overall weighted average of GRS A and B was calculated as 98.69%. Bhatt et al. discussed 4 and Butler et al. discussed 3 misplaced screws out of their 218 (1.8%) and 606 (0.49%) placed ones through the PS implantation, which were identified, replaced and revised intra-operatively [20]. Where the few GRS C or D PS grade were recognized through the post-operative follow-up (mainly in Lumbar 4 and 5 vertebrae), patients were 100% asymptomatic [22,25].

According to the screened publications, in 6 out of 7 centers (85.7%), the xVision Spine AR navigation system, approved by the U.S. Food and Drug Administration (FDA), was used by Augmedics Ltd. (Chicago, IL, USA) [29]. One center used an MR-based intra-operative three-dimensional image-guided navigation system (MITINS), including HoloLens by Microsoft (Redmond, WA, USA) [30]. For image–patient registration, xVision uses registration clamps and infrared light-reflecting optical markers, while MITNIS is based on electromagnetic tracking and navigation. No significant difference in clinical accuracy was recognized between the two systems.

### 3.3. Operation Time, Complications and Outcomes

Only Bhatt et al. presented specific data about operation time (OT) as an average of 3.6 ± 1.7 h. They added information on intra-operative blood loss of 224.0 ± 332.5 mL and the use of mean 3 packets of blood transfusion in their 32 patients. The length of hospital stay of their patients was 4.1 ± 1.6 days [20]. Specifically for PS insertion, Butler et al. added information about the average placement time of 3 min and 54 s per screw with a median of 4 min and 8 s (1 min 10 s to 6 min 30 s). They also measured the learning curve through their data collection, differentiating the experience of the first and final 20 patients’ procedures, where the mean insertion times were 4 min 1 s and 3 min 52 s per screw (*p* = 0.48) [24].

None of the 7 articles presented any surgical complications or the need for revision surgeries through the hospital stay or post-operative follow-up (from 2 weeks to 24 months). Clinical symptomatic reduction was noted in all patients through questionnaires by Yahanda et al., as well as significant improvement of VAS (18.4 ± 2.9) and ODI (16.4 ± 2.6) scores by Li et al. [21,23].

Bhatt et al. also added the mean total 3D imaging radiation dose for AR-navigated PS implantation, which was 576.8 ± 368.8 mGycm, and the average fluoroscopy time was 25.7 ± 29.8 s with a mean radiation dose of 0.3 ± 0.4 mGym^2^ [20].

### 3.4. Advantages and Limitations

Through the review, several comments were identified from the authors, as they expressed their experience with the use of AR-HMDs.

#### 3.4.1. Advantages

- Bhatt et al. [20] mentioned that the navigation system with AR technology is highly effective in real-world patient-care scenarios, without a significant learning curve needed for using it. He also added that, with just a limited disruption in workflow, AR-HMDs are simple to integrate, and with it, the implantation accuracy is elevated, and the overall radiation dose is decreased through the procedures compared with the FH technique.
- Yahanda et al. [23] commented on a similar or superior implantation accuracy of AR-HMDs compared with the most commonly used RASs (e.g., SpineAssist platform by Mazor, ExcelsiusGPS, ROSA or TianJi). He also added that the fluoroscopy time and radiation dose decreased through the surgeries.
- Liu et al. [22] added their accuracy and surgical workflow data to highlight the similarities with RASs too (Mazor X, ROSA, TianJi). It was noted as a strong benefit that using AR-HMDs minimizes the attention shift, as the user can simultaneously visualize the operation field and the image guidance too. With this, cognitive and motor task performance are increased. Another comment was that any instrument can be universally navigated with the AR-HMD system, causing only a minimum interruption in the line of sight. Additionally, the technology’s cost is not prohibitive to the distribution to patients.
- Harel et al. [25] described the collected data on their user experience questionnaire (UEQ, 1–7 numbered scale on 26 clinical usability questions) regarding the xVision system. All the scores were higher than 6 on average in the dimensions of the clarity of navigation display, the fit into the surgical workflow and the reliability of the system. The lowest score they noted was about the HMD ergonomics (5.9-point average).

#### 3.4.2. Limitations

- Li et al. [21] drew up the cost of AR-HMD systems compared to traditional FH/MIS methods. Additionally, they felt the disturbing defects of soft tissue simulation, the contrast of AR models, and images were limited by bright light and through the use of it, eye strain and visual discomfort may occur on the user, which needs training. For patients, unusually different laying posture was needed on the OR table because of the use of EM trackers and navigation tools.
- Liu et al. [22] commented on the limited use of the technology on obese patients (as the image–patient registration marker clamps needed to be fixed on rigid locations were too short, causing four patients to be excluded from the use case study). The disadvantages of the HMDs were described as mechanical and visual discomfort, visual obstruction and sensory overload, and lastly, the prevention of using headlights through the procedures.
- Yahanda et al. [23] gave the same notes about the learning curve of visual discomfort and disorientation of HMDs and the difficulties caused by the patient’s obesity.

### 3.5. Joint Analysis of All Available Studies

From the previous SLR by Burström et al., only one article was identified as eligible for our inclusion criteria. Abe et al. published a cohort study on their experience on vertebroplasty guided by the Epson Movero AR-headset in 2013 [18]. Altogether, five osteoporotic vertebra-fractured (Th.12–L3) patients were operated on without any pedicular breach (100% GRS A), having only 2.09° ± 1.3° axial and 1.98° ± 1.8° sagittal trajectory error of the 10 implanted screws. No complication was identified through their follow-up.

**Figure 3:**
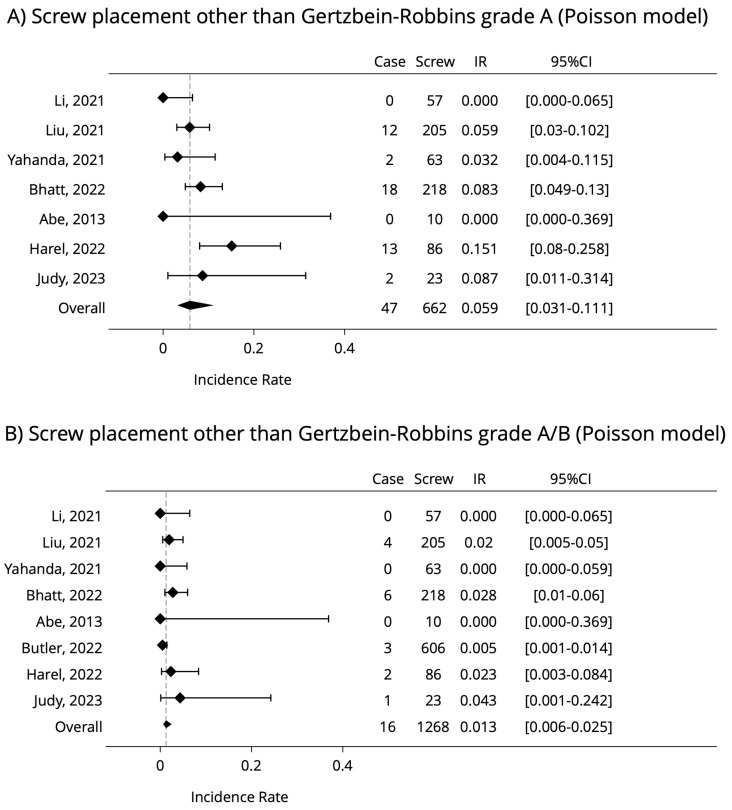
Results of multi-level Poisson model analysis on screw placement (the lower the better) [18,20,21,22,23,24,25,26].

As there were no complications described in either of the investigated articles, a meta-analysis could not be performed. For further analysis, a multi-level Poisson model was used for the hypothesis, which measures and explains the incidence rate of “screw error odds” for every single screw insertion [31]. The confidence intervals were calculated with the exact Poisson method [32]. The examination was made in two scenarios, highlighting only GRS A class and GRS A and B rates together, as those are still clinically completely acceptable. Figure 3 contains the results of the analysis.

**Figure 4:**
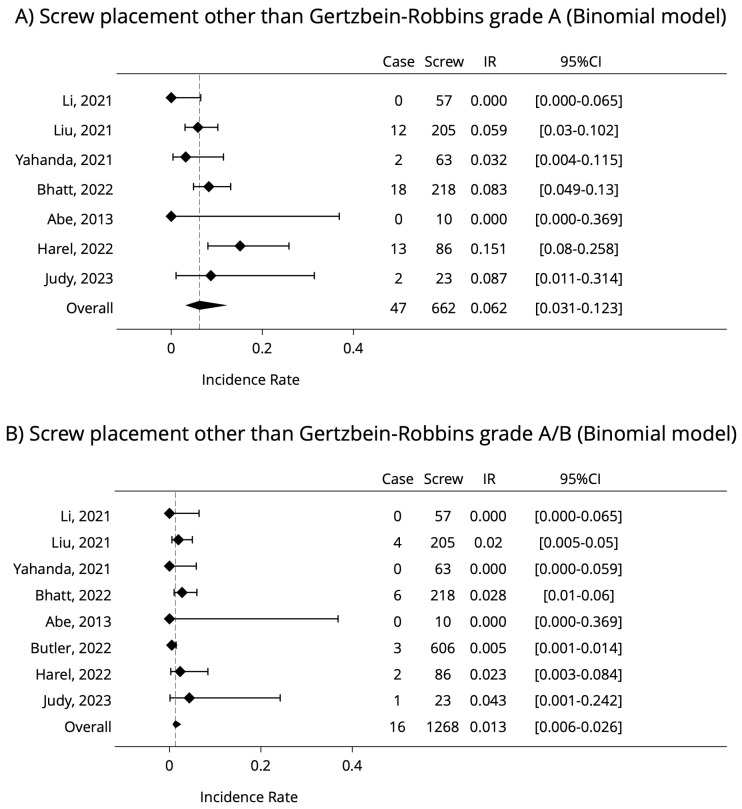
Results of multi-level binominal model analysis on screw placement (the lower the better) [18,20,21,22,23,24,25,26].

A multi-level binominal model was used in the ideology to measure the “screw error odds” for every single new screw insertion as further attempts for error. The confidence intervals were calculated with the binomial (Clopper–Pearson) method [33]. The same two scenarios were used as given above; Figure 4 shows the results.

In conclusion, non-GRS A may occur in 7.0% (95%CI: 2–11%), non-GRS A and B (clinically unacceptable grades) may occur in 1.2% (95%CI: 1–3.5%) of all the screw implantations.

## 4. Discussion

As highlighted from this review, standardized studies and reports for AR-HMDs navigated spine surgeries cannot yet be found in great numbers, the examined articles are greatly heterogeneous in describing objective outcomes. However, the authors presented evidence about the benefit of using this system compared to free-handed screw insertion. The reviewed recent studies discussed low case-numbered results, and no exact complication rates could be collected. A further meta-analysis may not be accurately performed and would identify a high level of risk of bias.

The authors strongly suggest that future studies and reports on the topic should be planned to contain standardized clinical, accuracy and procedural data for living patient care too. Linear tip errors and angular trajectory errors are measured only in phantom and cadaver studies, but those would include statistically more objective values for in vivo use rather than the clinically used Gertzbein–Robbins Scale. It is also clear that the relevance of such measurements in the clinical field is not intense, as, clinically, the main goal is to reduce pain and stabilize the spinal column or treat the morbidity without causing any neurological complications. We encourage researchers to consider our eligibility criteria (2.2.4 point) in the design of future studies. It would be desirable to develop points to consider for conducting clinical trials and observational studies in AR-HMDs guided spine surgery, specifying basic requirements, such as for study design and end points.

Both of this article’s multi-level statistical models resulted in approximately the same outcome, without significant differences from each other, showing that using AR-HMDs for spine surgery has only 1.2% GRS C-D-E grade. This value supports the hypothesis that the technology reached a higher clinical readiness level, as it confirms the existing knowledge in this research topic. According to the reviewed articles, the integration of this new technology was easy and time efficient, did not disrupt the clinical workflow, and all the clinical outcomes are similar or better compared to robot-assisted spine surgeries, which might make the AR-HMDs a cost-saving alternative method [15]. Applying to the surgical workflow, the AR-HMD system would not elongate significantly the operation time as well, which would elevate the risk of surgical site infection [34]. For future advancements, some recommendation was also mentioned: HMD-built-in light source, magnification lens and the system’s complete integration with RAS.

User satisfaction was clear in real-world scenarios; the system increased the pedicle screw placement accuracy and decreased the overall radiation dose needed for screw implantation. However, visual discomfort and eye strain may happen through use, and the use of AR-HMD guidance has limited possibilities for obese patients. Further development should take into account the importance of ergonomics and the comfort of long usability.

As seen across various domains, the recent pandemic accelerated the adaption of robotics in telemedicine and surgery as well [35]. One great advancement in the research topic was the FDA clearance of xVision by Augmedics [29]. According to the FDA regulatory clearance, the requirement was a mean position accuracy error of under 2 mm and a mean trajectory error of 2° for the new system based on X-ray imaging. In the investigation, the overall system accuracy, image registration accuracy and tracking accuracy were tested. Technical performance, user need and software validation, electrical safety and electromagnetic compatibility, headset cleaning, disinfection, reusability, and biocompatibility tests also passed [36]. These preferences could stand as industry standards for future surgical-use AR-HMDs development, and moreover, it could spin out to collateral domains, such as smart farming, where the revolution via the internet-of-everything concept has already begun [37], incorporating the sustainability aspects of such innovation programs [38]. The community has already started to align with these requirements (e.g., https://www.sustainablerobotics.org/ accessed on 1 May 2023).

Nevertheless, the ethical and regulatory aspects of the technology have to be managed in parallel to the technical advancement [39]. It is crucial to improve the transparency of the regulatory environment of AR/XR and RAS in medicine, streamline the standardization framework and increase the social acceptance, which is currently served by the standard family IEEE 700X (ethicsinaction.ieee.org/p7000 accessed on 1 May 2023), primarily to the IEEE 7000-2021-Model Process for Addressing Ethical Concerns During System Design [40]. Beyond this, the most recent IEEE 7007-2021 Ontologies for Ethically Driven Robotics and Automation Systems can also contribute to the field, which will be applied to the digital surgery domain as well.

## 5. Conclusions

The statistical analysis of the reviewed articles using AR-HMDs guidance on Pedicle Screw insertion in spine surgery showed the occurrence of 1.2% (95%CI: 1–3.5%) non-GRS A and B (clinically unacceptable grades) of all the screw implantations. The benefit of the system is clearly measurable compared to the free-handed implantation technique, yet the heterogeneity of published data prevents further meta-analysis. The authors used multi-level Poisson and binominal models for statistical analysis, and the results strongly support the claims of reviewed articles, that using the AR-HMD is as accurate as using the more expensive robot-assisted surgical systems.

Following a standardized methodology for future case studies or randomized clinical trials would help with a low-bias statistical analysis. Further development of the AR-navigated surgical systems is needed based on the experience of end users, aiming specifically for use in operating rooms.

## Figures and Tables

**Table 2:** Clinical accuracy of AR/MR-HMD (Augmented-/Mixed Reality Head-Mounted Display) navigated pedicle screw insertion. N/A Not Added.

Author	Year	Use Case	Nr. Cases	Nr. Screws	Linear Tip Error (mm)	Angular Trajectory Error (°)	Gertzbein–Robbins Scale	Device
Li, J. et al. [21]	2021/03	in vivo	7	57	N/A	N/A	100% A	MITINS system (HoloLens)
Liu, A. et al. [22]	2021/10	in vivo	28	205	N/A	N/A	94% A, 4% B	xVision, Augmedics
Yahanda, A.T. et al. [23]	2021/08	in vivo	9	63	N/A	N/A	96.8% A, 3.2% B	xVision, Augmedics
Bhatt, F.R. et al. [20]	2022/01	in vivo	32	218	N/A	N/A	97.1% A and B (4 misplaced screw revised intra-operatively)	xVision, Augmedics
Butler, A.J. et al. [24]	2022/09	in vivo	165	606	N/A	N/A	99.51% A and B (3 screws were replaced intra-operatively)	xVision, Augmedics
Harel, R. et al. [25]	2022/05	in vivo	19	86	N/A	N/A	97.7% A and B	xVision, Augmedics
Judy, B.F. et al. [26]	2023/01	in vivo	12	23	N/A	N/A	95.6% A and B	xVision, Augmedics

## Data Availability

Not applicable.

## Abbreviations

The following abbreviations are used in this manuscript:

AI	Artificial Intelligence
ATE	Angular Trajectory Error
CIS	Computer-Integrated Surgery
FDA	U.S. Food and Drug Administration
FH	Free Handed (vertebral procedure)
GRS	Gertzbein–Robbins Scale
HMD	Head-Mounted Display
IGS	Image-Guided Surgery
LTE	Linear Tip Error
MIS	Minimal Invasive Surgery
OT	Operation Time
PS	Pedicle Screw(ing)
RAS	Robot-Assisted Surgery
SLR	Systematic Literature Review
UEQ	User Experience Questionnaire
VR/AR/MR	Virtual/Augmented/Mixed Reality

## Appendix A

Search terms and search strategy for the SLR:

All papers published until 1 June 2022, with inclusion restriction of publication date after 27 November 2020.

PubMed

((((spine) OR (spinal)) AND (surgery)) OR (pedicle screw)) AND ((augmented reality) OR (mixed reality)).

Scopus

TITLE-ABS-KEY ((((spine OR spinal) AND surgery) OR (pedicle AND screw)) AND ((augmented AND reality) OR (mixed AND reality))).

Embase

(((spine OR spinal) AND surgery) OR (pedicle screw)) AND ((augmented reality) OR (mixed reality)).

IEEE Xplore

(((spine OR spinal) AND surgery) OR (pedicle screw)) AND ((augmented reality) OR (mixed reality)).

Cochrane Library

(((spine OR spinal) AND surgery) OR (pedicle screw)) AND ((augmented reality) OR (mixed reality)).
